# A Novel Variant in *TUBB4B* Causes Progressive Cone‐Rod Dystrophy and Early Onset Sensorineural Hearing Loss

**DOI:** 10.1002/mgg3.70068

**Published:** 2025-01-29

**Authors:** Margherita Scarpato, Francesco Testa, Anna Nesti, Roberta Zeuli, Rosa Boccia, Gennaro Auletta, Sandro Banfi, Francesca Simonelli, Marianthi Karali

**Affiliations:** ^1^ Medical Genetics, Department of Precision Medicine University of Campania ‘Luigi Vanvitelli’ Naples Italy; ^2^ Multidisciplinary Department of Medical, Surgical and Dental Sciences, Eye Clinic University of Campania ‘Luigi Vanvitelli’ Naples Italy; ^3^ Department of Neuroscience, Reproductive Science and Dentistry University of Naples Federico II Naples Italy; ^4^ Telethon Institute of Genetics and Medicine Pozzuoli Italy

**Keywords:** progressive cone‐rod dystrophy, sensorineural hearing loss, syndromic inherited retinal disease, *TUBB4B*

## Abstract

**Background:**

Sensorineural hearing loss (SNHL) is a frequent manifestation of syndromic inherited retinal diseases (IRDs), exemplified by the very rare form of autosomal‐dominant Leber congenital amaurosis with early onset deafness (LCAEOD; OMIM #617879). LCAEOD was first described in 2017 in four families segregating heterozygous missense mutations in *TUBB4B*, a gene encoding a β‐tubulin isotype. To date, only eight more families with similar *TUBB4B*‐associated sensorineural disease (SND) have been reported. Most cases harbored missense variants affecting the same amino acid (Arg391) and only three families segregated variants involving different residues (Tyr310, Arg390).

**Methods:**

We performed whole‐exome sequencing and a full ophthalmological and audiological examination of the affected members in an Italian family segregating syndromic IRD with early onset deafness.

**Results:**

We identified a novel, ultra‐rare, disease‐causing variant in *TUBB4B* (NM_006088.6:c.1049A>C) that replaces a highly conserved lysine with threonine at amino acid position 350. The functional impact of the Lys350Thr substitution was supported by protein structure modeling studies. The variant segregates in the family members presenting retinal disease with early onset SNHL. Detailed ophthalmological assessment of the affected subjects diagnosed a progressive cone‐rod dystrophy.

**Conclusion:**

These findings expand the limited number of disease‐causing *TUBB4B* variants, corroborating their association with SND forms, and suggest Lys350 is an important residue for β‐tubulin function. Interestingly, our results demonstrate that *TUBB4B* mutations can cause cone‐dominated retinal phenotypes.

## Graphical abstract

Whole‐exome sequencing of affected members in a family segregating dominant syndromic retinal disease with early onset deafness revealed a novel, ultra‐rare, disease‐causing variant in *TUBB4B* (NM_006088:c.1049A>C). The variant substitutes a highly conserved lysine with threonine at amino acid position 350. Protein structure modeling studies suggest that Lys350 is an important residue for β‐tubulin function, and detailed ophthalmological analyses demonstrate that the Lys350Thr TUBB4B variant causes progressive cone dystrophy with early onset sensorineural hearing loss.

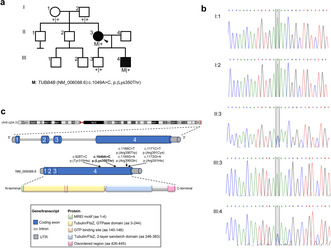

## 1 Introduction

Inherited retinal diseases (IRDs) are genetically heterogeneous monogenic diseases characterized by progressive degeneration or dysfunction of photoreceptor cells. Their clinical manifestations are variable, ranging from isolated retinal dysfunction to complex syndromes. Sensorineural hearing loss (SNHL) is the most recurrent extraocular feature in syndromic IRD forms (Tatour and Ben‐Yosef 2020), in part due to the shared structural and functional properties of the light‐ and sound‐sensory receptor cells in the retina and inner ear, respectively. The most common syndromic IRD is Usher syndrome, which is characterized by retinitis pigmentosa (RP) and SNHL with variable degrees of vestibular involvement. Besides Usher, in which the vision defects initiate with the dysfunction of rod photoreceptors, additional monogenic forms of combined deaf‐blindness with primary cone involvement have been described (Namburi et al. 2016; Nikopoulos et al. 2016). Both rod‐ and cone‐dominated forms of deaf‐blindness often result from defects in genes encoding key proteins of ciliary development and function, which, when mutated, disrupt proper formation and function of ciliated cells in the retina and inner ear (Falk et al. 2015).

Tubulins are key components of ciliary function for their role in microtubule assembly and cytoskeleton dynamics. *TUBB4B* encodes a ubiquitously expressed, cilia‐specific isotype of *β*‐tubulin, which is particularly abundant in the retina, brain, testis, and bone marrow (Luscan et al. 2017; http://www.proteinatlas.org/ENSG00000188229‐TUBB4B/tissue) and is essential for proper centriole and axoneme formation in ciliated cells of different tissues (Dodd et al. 2024; Sewell, Legué, and Liem 2024). Mutations in *TUBB4B* disrupt the assembly and function of motile and sensory cilia through dominant‐negative mechanisms, leading to ciliopathic phenotypes with syndromic manifestations (Dodd et al. 2024; Sewell, Legué, and Liem 2024). Despite its widespread expression, *TUBB4B* has been implicated in three distinct classes of autosomal‐dominant ciliopathic disease in humans: (a) in primary ciliary dyskinesia (PCD), (b) in a sensorineural disease (SND) that presents with Leber congenital amaurosis and early onset hearing loss (LCAEOD; OMIM #617879), and (c) in their combination (PCD + SND). These three disease forms arise, respectively, from the dysfunction either exclusively of motile cilia or of sensory cilia only or of both cilia types (Dodd et al. 2024). Variant‐to‐function studies demonstrated that the distinct clinical manifestations depend on the β‐tubulin interface affected by each variant and on the extent the mutation interferes with microtubule dynamics in a given tissue (Dodd et al. 2024).

Reports on disease‐causing variants in *TUBB4B* are limited, with the identification of missense changes involving only six amino acid residues—namely, Gln11, Tyr310, Pro259, Pro358, Arg390, and Arg391—each associated with distinct disease phenotypes (Luscan et al. 2017; Dodd et al. 2024; Sewell, Legué, and Liem 2024; Bodenbender et al. 2024; Maasz et al. 2022; McFadden et al. 2023). Of those, only missense variants at amino acid positions 391 (Arg391His, Arg391Cys), 390 (Arg390Gln), and, more recently, 310 (Tyr310His) have been linked to the isolated SND (i.e., without PCD) (Luscan et al. 2017; Dodd et al. 2024; Bodenbender et al. 2024; Maasz et al. 2022). The retinal phenotypes associated with these variants were in most cases indicative of a generalized photoreceptor dysfunction similar to Leber congenital amaurosis (LCA) (Luscan et al. 2017; Dodd et al. 2024; Bodenbender et al. 2024; Maasz et al. 2022), but atypical rod‐dominated forms, such as pericentral RP, as well as a milder sectorial RP in a patient with mosaicism for the pathogenic variant, have also been reported (Bodenbender et al. 2024).

Here, we report a novel disease‐causing, missense variant in *TUBB4B* (NM_006088.6:c.1049A>C) predicted to substitute a highly conserved lysine with threonine at amino acid position 350 in two affected individuals with early onset progressive cone‐rod dystrophy and hearing loss. This variant adds up to the few missense changes (affecting only three other amino acid residues) described thus far to be involved in *TUBB4B*‐related SND and extends the ophthalmological phenotype associated with this condition. These findings can have implications for the genetic diagnosis of patients with syndromic IRD forms and can contribute to a better understanding of the molecular mechanisms underlying the onset of specific sensory defects in these tubulinopathies.

## 2 Materials and Methods

### 2.1 Ethical Compliance

All procedures were conducted in accordance with the tenets of the Declaration of Helsinki and received approval from the Ethics Board of the University of Campania “Luigi Vanvitelli”. Written informed consent was obtained by the proband and her relatives for participation in the study.

### 2.2 Ophthalmological Assessment

The ophthalmological examination included measurements of best‐corrected visual acuity (BCVA), slit lamp anterior segment examination, intraocular pressure, color vision testing, fundus examination, spectral domain optical coherence tomography (SD‐OCT), fundus autofluorescence imaging (FAF), and full‐field electroretinography (ERG). SD‐OCT and FAF imaging were performed with a Heidelberg Eye Explorer Version 1.9.11.0 (Heidelberg, Germany). ERG recordings followed the international guidelines established by the International Society for Clinical Electrophysiology of Vision (ISCEV) (Marmor et al. 2009).

### 2.3 Audiological Assessment

The audiological evaluation comprised otoscopy, pure‐tone audiometry covering frequencies from 125 to 8000 Hz for air conduction and from 250 to 4000 Hz for bone conduction, with the determination of the pure tone average (PTA), auditory brainstem response, and other pertinent audiological data. PTA was calculated considering the thresholds at 500, 1000, 2000, and 4000 Hz. The severity of hearing loss was categorized based on PTA according to the World Health Organization classification system: mild (PTA 25–40 dB), moderate (PTA 41–60 dB), severe (PTA 61–80 dB), and profound (> 80 dB).

### 2.4 Molecular Genetic Testing

Peripheral blood samples were collected following written informed consent. Genomic DNA extraction was performed using the DNeasy Blood & Tissue Kit (QIAGEN) following standard protocols. Whole‐exome libraries were prepared using the SureSelect technology (Human All Exon v7, Agilent, Santa Clara, CA, USA) and sequenced on the NovaSeq 6000 Sequencing System (Illumina Inc., San Diego, CA, USA). Variant calling and annotation were conducted using a previously described pipeline (Di Iorio et al. 2017). Only variants with a minor allele frequency (MAF) < 0.01 were retained and prioritized based on variant type, predicted protein effect, and in silico pathogenicity scores. Read alignments at candidate positions were visually inspected using the Integrative Genomics Viewer (IGV). Selected variants were validated by Sanger sequencing of the corresponding genomic fragments, and segregation analysis was performed in additional family members.

### 2.5 Protein Structure Modeling of the Identified Variant

Homology modeling was employed to predict the impact of the Lys350Thr substitution on the structure, stability, and dynamics of the TUBB4B protein. The human TUBB4B‐TUBA1A heterodimer structure, resolved at 2.8 Å resolution by electron microscopy, was retrieved from the Protein Data Bank (PDB code: 8SH7) and used as template for the homology model (Kellogg et al. 2018). The homology model of the TUBB4B protein containing the Lys350Thr substitution was generated using the DynaMut2 web server (Rodrigues, Pires, and Ascher 2021). DynaMut2 employs advanced molecular dynamics simulations and machine learning techniques to predict the impact of amino acid substitutions on protein stability, flexibility, and dynamics. The software considers factors such as changes in hydrogen bonding, electrostatic interactions and conformational entropy to provide a comprehensive assessment of the structural and energetic consequences of the mutation.

### 2.6 Protein Alignments

Protein sequences of TUBB4B orthologs across multiple species, as well as the sequences of all human alpha and beta tubulin isoforms, were obtained from the UniProt database. Sequences were aligned using the Clustal Omega algorithm (Sievers et al. 2011) with default parameters via the UniProt Align tool to assess the evolutionary conservation of the Lys350 residue and its surrounding region.

## 3 Results

### 3.1 Clinical Presentation of the Reported Cases

**FIGURE 1:**
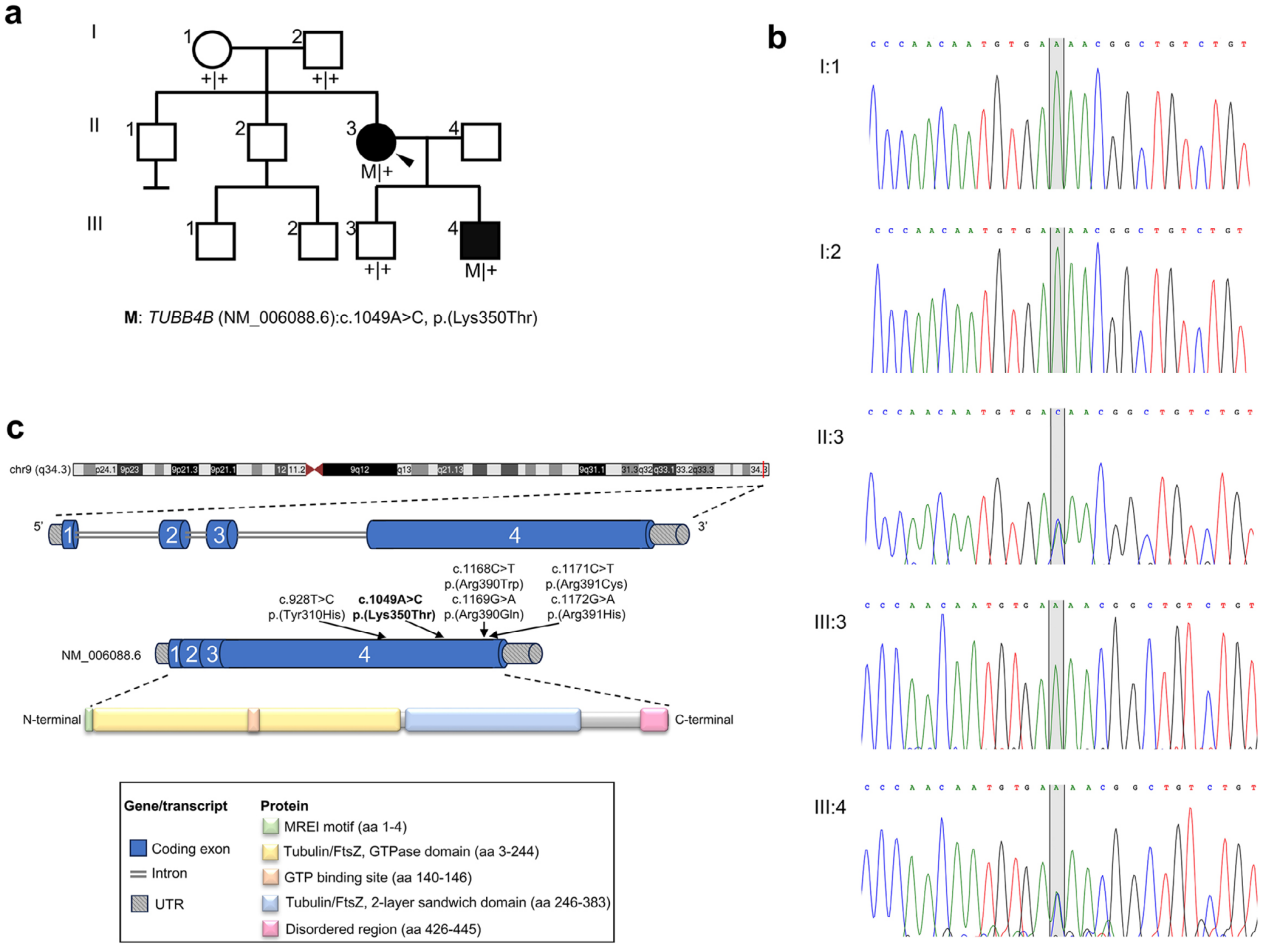
Genetic findings in the affected individuals. (a) Pedigree and variant (M) segregation in the reported family. Filled symbols denote affected individuals. The proband (II.3) is indicated by an arrowhead. (b) Sanger sequencing chromatograms of the region spanning the *TUBB4B* (NM_006088.6):c.1049A>C variant (shaded in grey) from the analyzed family members. The proband (II:3) and her affected son (III:4) are heterozygous for the variant (A/C), whereas her unaffected parents (I:1, I:2) and son (III:3) are homozygous for the reference sequence (A/A). (c) Schematic illustration (not to scale) of the *TUBB4B* gene and protein structure showing the position of the genetic variants identified to date in cases with sensorineural disease (the identified missense variant is given in bold).

A Caucasian female subject was referred to our clinic at 23 years of age with complaints of poor vision complicated by early onset hearing impairment. She reported onset of visual symptoms at 6 years with reduction of central visual acuity and concomitant onset of hearing loss. Her medical history included an episode of encephalitis following a rubella virus infection and a diagnosis of pituitary microadenoma at 22 years. The proband was born to nonconsanguineous, healthy parents who did not report any vision or hearing difficulties, as confirmed by a full ophthalmological and audiological checkup at their late 40s. She had two unaffected brothers and there were no reports of other affected members in the maternal and paternal sides of the family (Figure 1a). The proband had two sons, one who suffered from similar symptoms and one unaffected (Figure 1a).

The proband was seen at our clinic for the first time at the age of 23 years and subsequently performed regular follow‐up controls for over two decades. At the first ophthalmological observation, she referred photophobia since the age of 6 years and her BCVA was 20/100 in both eyes with bilateral myopia of 2 diopters (D). The lens and cornea appeared clear under biomicroscopy in both eyes, and intraocular pressure was normal. She presented achromatopsia, ascertained by the Ishihara color vision test. In full‐field ERG, scotopic responses were normal, whereas the photopic ones were below the noise level. On the basis of the above findings, she was diagnosed with cone dystrophy.

**FIGURE 2:**
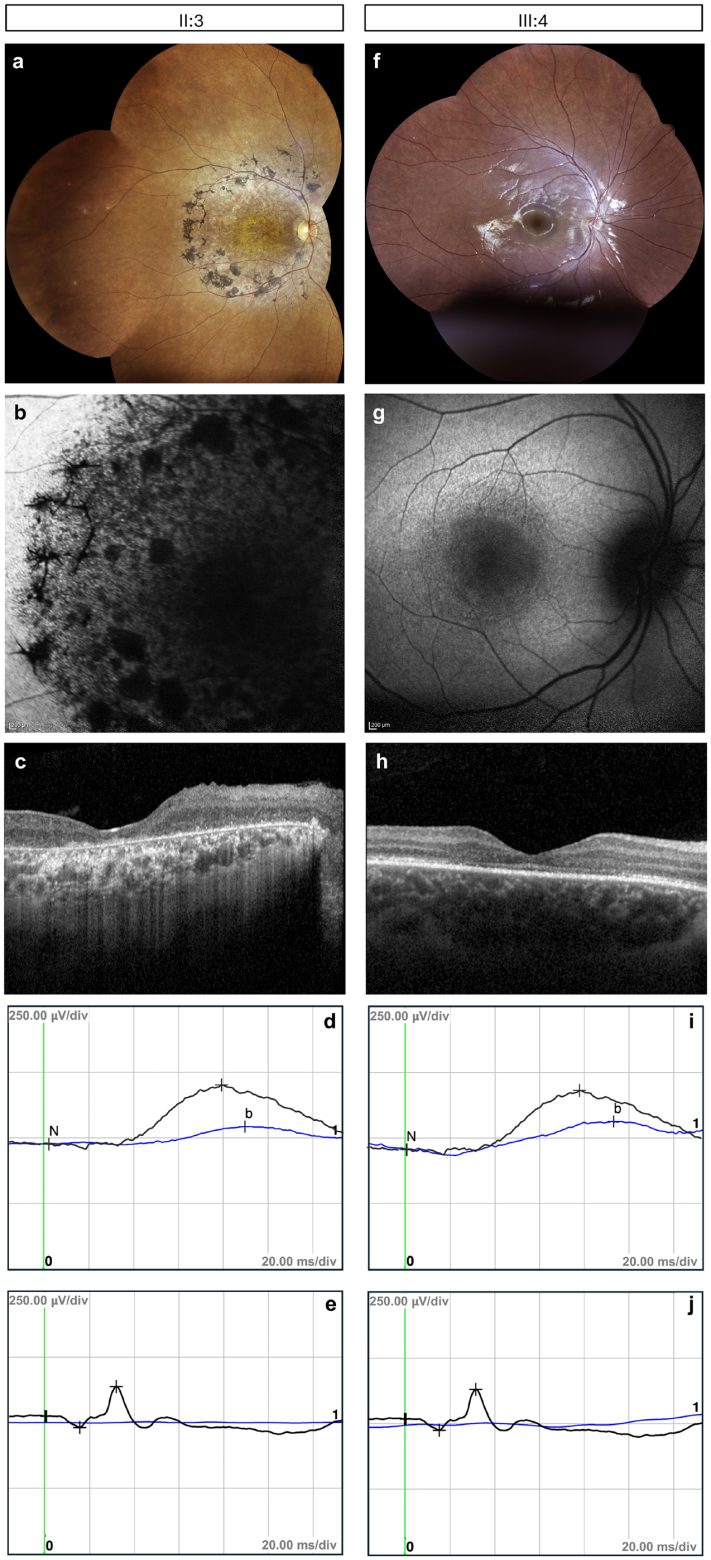
Ophthalmological findings in the proband and her affected son. (a, f) Fundus imaging of the affected individuals. The proband (II:3) presented a quite pale optic disc, reduction of retinal vessel size, areas of RPE dystrophy at the posterior pole, and pigment deposits (bone‐spicule like and clumps) primarily distributed along the vascular arcades. No pigment deposits were visible in the far periphery and in the central/macular area (a). The proband's affected son (III:4) had a normal fundus, without pigment deposits or evident signs of RPE dystrophy (f). (b, g) Fundus autofluorescence (FAF) imaging. Reduced autofluorescence (AF) and focal areas of definitely decreased AF (DDAF) at the posterior pole were observed in the proband (b). Her son presented an overall normal AF distribution at the posterior pole, with fine stippling of hypoautofluorescent spots around the macular area (g). (c, h) Optical coherence tomography (SD‐OCT) imaging. SD‐OCT showed reduced macular thickness, RPE dystrophy/atrophy, and vitreoretinal interface syndrome in the proband (c). In her son, a subtle reduction of macular thickness and discontinuous/disrupted ellipsoid zone (EZ) band at the foveal region (red arrows) was observed (h). (d, e, i, j) Scotopic and photopic ERG responses. Patient ERG traces are depicted in blue and normal reference traces in black. The amplitudes of the scotopic ERG responses were subnormal in the proband (approximately 30% lower than the norm) (d) and normal in her son (i), whereas the photopic responses were unrecordable in both (e, j).

The follow‐up assessments indicated the progressive nature of the disease. Briefly, the BCVA decreased steadily over time from “count finger” in both eyes at 30 years to “hand motion” perception from 39 years onward. The decrease in BCVA was further accentuated by the onset of nystagmus at 30 years of age. Fundus changes were also observed during the follow‐up. Retinal pigment epithelium (RPE) dystrophy appeared at the posterior pole at the age of 30 years. At 39 years of age, both typical (bone‐spicule like) and atypical (clumps) pigment deposits were visible also in mid‐periphery, scattered primarily along the vascular arcades, but sparing the far periphery as it is typically observed in pericentral RP (Karali et al. 2019). This pattern of pigment distribution was confirmed at the most recent fundus inspection at 46 years (Figure 2a). The involvement of the central retina was more evident in FAF images that showed reduced FAF with focal areas of definitely decreased FAF (DDAF) at the posterior pole, indicative of RPE dystrophy/atrophy at the macular area (Figure 2b). SD‐OCT imaging revealed morphological changes in the retinal structure, such as thinning of the central retina, no detectable ellipsoid zone (EZ) band, and atrophy of the RPE (Figure 2c). The retinal morphology was further compromised at the age of 35 years, when the patient developed a vitreoretinal interface syndrome. Retinal dysfunction exacerbated over time, affecting also the rod‐mediated responses. The scotopic ERG responses, which were initially normal, were still recordable at the latest assessment albeit subnormal (approximately 30% lower than the normal value) (Figure 2d), whereas the photopic ones were not detectable (Figure 2e). The lens and cornea remained clear and the myopia stable in both eyes till the latest assessment. On the basis of the above observations, the cone‐rod dystrophy was defined as progressive.

The proband complained of prelingual hearing loss, with self‐reported onset in early childhood, which had impacted her language development. She also suffered from catarrhal otitis, which was initially suspected to be the cause of the hearing loss. She had no complaints of tinnitus or dizziness. Audiological evaluation diagnosed bilateral, preverbal SNHL of a moderate grade, with a PTA of 78 and 52 dB in the right ear and left ear, respectively (data not shown). Impedance testing produced a normal Type A tympanogram bilaterally with stapedial reflexes in *ipsi* and *contra* across the entire frequency range, suggesting normal middle ear function. The patient was fitted with hearing aids at childhood and underwent regular audiological checkups.

The proband's second child was also affected (Figure 1a). He was born at term by caesarean delivery after an uncomplicated pregnancy and his auxological parameters were within the normal range. At the newborn hearing screening, he was diagnosed with bilateral SNHL and was fitted with hearing aids at 5 months of age. His speech and language development were normal from the age of 3 years onward. His psychomotor development was also normal, and he did not present any systemic problems or abnormalities. At the age of 7 years, he underwent a full ophthalmological examination following complaints of reduced central visual acuity with onset at preschool age. At the latest ophthalmological assessment (at the age of 9 years), he had a BCVA of 20/200 in both eyes and mild hypermetroptic astigmatism with a clear lens and cornea. The retinography revealed an almost normal fundus, without evident signs of RPE dystrophy or pigment deposits (Figure 2f). FAF imaging showed an overall normal distribution of AF at the posterior pole, with a fine granular pattern of hypoautofluorescent spots (stippling) surrounding the macular area (Figure 2g). SD‐OCT showed a mild reduction of macular thickness and a discontinuous/disrupted EZ band at the foveal region (Figure 2h), which is consistent with the reduced visual acuity of the subject. The scotopic ERG responses were normal (Figure 2i), whereas the photopic ones were subnormal (Figure 2j). These findings suggested a cone dystrophy, presumably progressive (given the maternal phenotype), whose progression should be assessed over time. The clinical findings in the affected individuals are mapped to Human Phenotype Ontology (HPO) terms in Table S1.

### 3.2 Genetic Testing Identified a Novel Variant in *TUBB4B*

**FIGURE 3:**
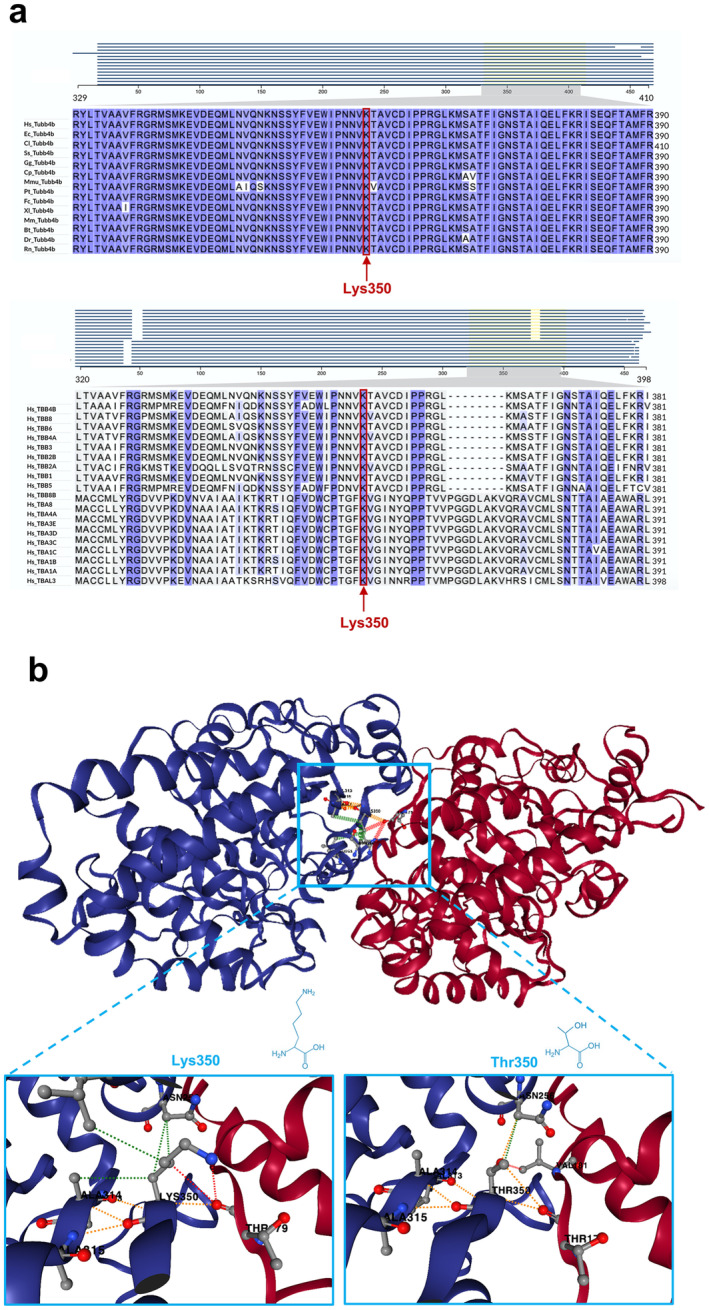
Protein alignment of TUBB4B vertebrate orthologues at the location of the identified variant and 3‐D modeling of the mutated TUBB4B. (a) Protein alignment of TUBB4B orthologues from vertebrates at the location of the identified variant showing high conservation of Lys350 (upper panel), and protein alignment of all human α‐ and β‐tubulin isoforms (lower panel). The intensity of the violet shading indicates the percentage identity of the amino acids at each position. The highly conserved lysine at amino acid position 350 of human TUBB4B is indicated by a red arrow. Amino acid sequences were retrieved from the UniProt database. The alignments were performed using the Clustal Omega algorithm with default parameters in the UniProt Align tool. In the upper panel, the accession numbers of the TUBB4B sequences from different species are as follows: *Homo sapiens* : Hs_Tubb4b, P68371; *Equus caballus* : Ec_Tubb4b, F6TLD9; *Canis lupus familiaris* : Cl_Tubb4b, A0A8C0MG20; *Sus scrofa* : Ss_Tubb4b, A0A8D0XJF4; *Gallus gallus* : Gg_Tubb4b, A0A8V1AEW4; *Cavia porcellus* : Cp_Tubb4b, H0VKM7; *Macaca mulatta* : Mmu_Tubb4b, I0FPW0; *Pan troglodytes* : Pt_Tubb4b, K7CDA3; *Felis catus* : Fc_Tubb4b, M3W0Z9; *Xenopus laevis* : Xl_Tubb4b, P30883; *Mus musculus* : Mm_Tubb4b, P68372; *Bos taurus* : Bt_Tubb4b, Q3MHM5; *Danio rerio* : Dr_Tubb4b, Q6P5M9; and *Rattus norvegicus* : Rn_Tubb4b, Q6P9T8. In the lower panel, the accession numbers of the human α‐ and β‐tubulin isoforms are as follows: Hs_TBB4B, P68371; Hs_TBB8, Q3ZCM7; Hs_TBB6, Q9BUF5; Hs_TBB4A, P04350; Hs_TBB3, Q13509; Hs_TBB2B, Q9BVA1; Hs_TBB2A, Q13885; Hs_TBB1, Q9H4B7; Hs_TBB5, P07437; Hs_TBB8B, A6NNZ2; Hs_TBA8, Q9NY65; Hs_TBA4A, P68366; Hs_TBA3E, Q6PEY2; Hs_TBA3D, P0DPH8; Hs_TBA3C, P0DPH7; Hs_TBA1C, Q9BQE3; Hs_TBA1B, P68363; Hs_TBA1A, Q71U36; and Hs_TBAL3, A6NHL2. (b) Three‐dimensional structure of human TUBB4B/TUBA1A heterodimer represented as a cartoon with α‐tubulin in red and β‐tubulin in blue. The effect of the Lys350Thr substitution on protein stability and dynamics was predicted for the 8SH7 PDB structure of the TUBB4B/TUBA1A heterodimer using the DynaMut2 web server. The light blue framed area shows the position of the TUBB4B Lys350 at the heterodimer interface (upper panel). The predicted effect of the amino acid substitution on the interface of the αβ‐tubulin heterodimer and the sidechain interactions are shown in the lower panel (Lys350 on the left side and Thr350 on the right side). Dotted lines indicate hydrogen bonds (red), hydrophobic interactions (green), and polar interactions (orange).

To identify the genetic cause of the sensory impairment in the proband and her son, a duo whole‐exome sequencing (WES) analysis was performed on genomic DNA. After variant filtering and prioritization, we identified a heterozygous point mutation in the *TUBB4B* gene (NM_006088.6) that was shared between the two affected subjects. Segregation analysis in the proband's unaffected son and parents confirmed the proper segregation of the variant with the sensory impairment phenotype (Figure 1a,b). The identified point mutation (NM_006088.6:c.1049A>C) is predicted to cause the substitution of a highly conserved lysine with threonine at amino acid position 350 of the encoded tubulin beta 4B (UniProt P68371) (Figures 1c and 3a). This missense variant was novel; it had not been previously reported in literature, in population databases (e.g., gnomAD genomes/exomes, Regeneron Pharmaceuticals dataset, http://rgc‐research.regeneron.com/me/gene/TUBB4B) or in repositories of genomic variation (e.g., ClinVar, the Human Gene Mutation Database [HGMD], and the Leiden Open Variation Database [LOVD]). Moreover, in our internal database of genomic variants from over 7524 WES or clinical exomes from Italian patients, the c.1049A>C variant was only detected in the two affected subjects described herein (Karali et al. 2022). According to the American College of Medical Genetics and Genomics (ACMG) recommendations, the missense variant p.(Lys350Thr) was classified as “likely pathogenic” based on the PP3, PM2, and PP2 criteria. The variant had a Combined Annotation Dependent Depletion (cadd; http://cadd.gs.washington.edu) score of 27 and a MutScore of 0.572 (http://mutscore‐wgt7hvakhq‐ew.a.run.app/; Quinodoz et al. 2022), and was predicted to be pathogenic by the majority of in silico pathogenicity prediction tools.

### 3.3 Structural Predictions of the Impact of the Identified Missense Variant

TUBB4B is a β‐tubulin isotype composed of 445 amino acids organized in a typical αβ‐class protein, formed by 12 α‐helices and 10 β‐strands. The lysine affected by the variant reported herein (Lys350) is located within the Tubulin/FtsZ two‐layer sandwich domain, at the interface region of the TUBB4B‐TUBA1A heterodimer, where it contributes to the stability of the complex through electrostatic interactions and hydrogen bonds with adjacent residues (Figure 3b). Protein alignment of TUBB4B vertebrate orthologues from 14 species showed that the lysine at position 350 is extremely conserved (Figure 3a, upper panel). Moreover, alignment of all human α‐ and β‐tubulin isoforms revealed a high degree of amino acid similarity at position 350 across all isotypes (Figure 3a, lower panel). To predict the effect of the Lys350Thr substitution at its structural context, an in silico 3‐D modeling analysis was performed. The substitution of the highly conserved Lys350 with threonine introduces a smaller, hydrophobic, and neutral amino acid residue at the place of the original, positively charged lysine. This change is predicted to alter the chemical properties of TUBB4B and to interfere with the stability of the TUBB4B–TUBA1A heterodimer. Specifically, the Lys350Thr substitution is predicted to alter the multimeric contacts with the ligand, affecting both the type of side chain interactions and the amino acids involved in these chemical bonds (Figure 3b).

## 4 Discussion

In this study, we provide a detailed ophthalmological characterization of a 45‐year‐old female and her 9‐year‐old son harboring a novel missense variant in *TUBB4B*. Both subjects were diagnosed with cone dystrophy and bilateral SNHL with reported onset of symptoms in early childhood. Early ERG findings indicated the primary functional impairment of cone photoreceptors in both subjects. The long follow‐up of the proband enabled us to establish the progressive nature of the vision loss, which initiated with the hallmarks of cone dystrophy, transitioning to a cone‐rod dystrophy over time. The progression of the retinal degeneration was further evidenced by a reduction in visual acuity, onset of nystagmus, and accumulation of pigment at the posterior pole. The retinal phenotype of the two subjects exhibits subtle differences to what has been described for *TUBB4B*‐related SND cases, which either present a progressive panretinal dystrophy affecting both photoreceptor cell types or atypical rod‐dominated forms (such as pericentral RP) (Luscan et al. 2017; Dodd et al. 2024; Bodenbender et al. 2024; Maasz et al. 2022). Therefore, the cone‐dominated phenotype associated with the p.(Lys350Thr) mutation in this family underscores the heterogeneous retinal manifestations of *TUBB4B*‐related disorders. In that respect, our findings suggest that ERG assessments in patients with early onset SNHL are instrumental to uncover early stage photoreceptor dysfunction, even at subclinical stages, and to deliver accurate clinical diagnosis.

The genetic analysis of both subjects identified a shared missense variant in the *TUBB4B* gene that was classified as “likely pathogenic” based on the ACMG criteria and in silico predictions. The variant was not detected in the unaffected parents of the proband, suggesting a de novo event. In addition, its absence in her unaffected son confirmed proper segregation with the disease phenotype. This ultra‐rare variant (NM_006088.6:c.1049A>C) had not been previously documented in literature or in population databases and involved a different amino acid residue (Lys350) from the variants reported thus far, therefore representing a meaningful expansion of the mutational spectrum associated with *TUBB4B*‐related disorders. Intriguingly, a number of rare coding variants in the *TUBB4B* gene were recently shown to significantly correlate with left handedness in humans in exome‐wide association studies (Schijven et al. 2024). The pathogenic variants reported in patients with sensory phenotypes were not detected among those (Schijven et al. 2024), neither was the p.(Lys350Thr) identified in this study.

Our findings suggest that Lys350 is an important residue for β‐tubulin function. The p.(Lys350Thr) missense change potentially interferes with the chemical properties of TUBB4B because the substitution of the highly conserved lysine residue with threonine is expected to disrupt critical protein–protein interactions and to compromise the stability of the tubulin heterodimer, as indicated by structural predictions. Given the role of Lys350 in stabilizing the assembly of TUBB4B‐TUBA1A heterodimers, it is reasonable to speculate that the variant described here contributes to the onset of the syndromic IRD phenotype by perturbing microtubule dynamics in ciliated cells of the sensorineural system. Functional studies are warranted to assess the impact of this disease‐causing variant on tubulin function, microtubule dynamics, and ciliogenesis and to elucidate the molecular mechanism underlying the specific sensitivity of cones to this variant. The intriguing observation that in two *Chlamydomonas reinhardtii* β‐tubulin mutants, the Lys350 substitution with glutamic acid or methionine increased their resistance to microtubule inhibitors, possibly by enhancing microtubule stability, had already suggested the importance of this residue for β‐tubulin function (Schibler and Huang 1991; Lee and Huang 1990).

In conclusion, the identification of this novel variant can improve the genetic diagnosis of patients with syndromic IRD forms. Moreover, this study reinforces the complex genotype–phenotype relationship proposed for *TUBB4B*‐associated disorders (Dodd et al. 2024), particularly regarding the retinal manifestations. Finally, our findings emphasize the critical role of Lys350 in maintaining proper TUBB4B function within sensory neurons.

## Author Contributions

Margherita Scarpato and Francesco Testa equally contributed to the work and drafted the manuscript. Marianthi Karali and Francesca Simonelli designed and supervised the study, and critically revised the manuscript. Margherita Scarpato, Roberta Zeuli, Sandro Banfi, and Marianthi Karali contributed to the collection and analysis of genetic data. Francesco Testa, Anna Nesti, Rosa Boccia, Gennaro Auletta, and Francesca Simonelli contributed to the collection and analysis of clinical data. All authors have read and agreed to the published version of the manuscript.

## Conflicts of Interest

The authors declare no conflicts of interest.

## Supporting information


Table S1.

## Data Availability

The exome sequencing dataset is not publicly accessible due to patient privacy concerns. Data sharing requests may be considered on a case‐by‐case basis with appropriate ethical approval.
