# Supplementary material for: A Novel Variant in TUBB4B Causes Progressive Cone‐Rod Dystrophy and Early Onset Sensorineural Hearing Loss
Source: Mol Genet Genomic Med. 2025 Jan 29;13(2):e70068. doi: 10.1002/mgg3.70068 (PMC11775458; doi:10.1002/mgg3.70068)
Supplement: Supplementary file 1 — Table S1. [file MGG3-13-e70068-s001.pdf]

A novel variant in *TUBB4B* associates with progressive cone dystrophy and early-onset sensorineural hearing loss

Margherita Scarpato[1†], Francesco Testa[2†], Anna Nesti[2], Roberta Zeuli[1], Rosa Boccia[2], Gennaro Auletta[3], Sandro Banfi[1,4], Francesca Simonelli[2*], Marianthi Karali[1,2*]

[1] Medical Genetics, Department of Precision Medicine, University of Campania 'Luigi Vanvitelli', Via Luigi De Crecchio 7, 80138, Naples, Italy
[2] Multidisciplinary Department of Medical, Surgical and Dental Sciences, Eye Clinic, University of Campania 'Luigi Vanvitelli', Via Pansini 5, 80131, Naples, Italy
[3] Dept. of Neuroscience, Reproductive Science and Dentistry, University of Naples Federico II, Via Pansini 5, 80131, Naples, Italy
[4] Telethon Institute of Genetics and Medicine, Via Campi Flegrei 34, 80078, Pozzuoli, Italy

Supporting Information

Table S1. Clinical findings in the affected individuals mapped to Human Phenotype Ontology (HPO) terms

HPO Term Identifier	Description
HP:0000006	Autosomal dominant inheritance
HP:0000548	Cone/cone-rod dystrophy
HP:0003676	Progressive
HP:0000529	Progressive visual loss
HP:0007663	Reduced visual acuity
HP:0000613	Photophobia
HP:0011516	Achromatopsia
HP:0030465	Undetectable light-adapted electroretinogram non-detectable photopic ERG
HP:0030601	Abnormal posterior segment imaging
HP:0030602	Abnormal fundus autofluorescence imaging
HP:0030607	Reduced OCT-measured macular thickness
HP:0007703	Abnormality of retinal pigmentation
HP:0008619	Bilateral sensorineural hearing impairment
HP:0008527	Congenital sensorineural hearing impairment
HP:0008610	Infantile sensorineural hearing loss
HP:0000399	Prelingual sensorineural hearing impairment
